# Experience of the Burden of Using Multiple Medicines and the Associated Impact on Health-Related Quality of Life

**DOI:** 10.1177/23743735251330353

**Published:** 2025-03-21

**Authors:** Aastha Gurung, Edward Ogden, Won Sun Chen

**Affiliations:** 1 School of Health Sciences, 3783 Swinburne University of Technology, Hawthorne, Victoria, Australia; 2 Curtin Medical School, 1649 Curtin University, Bentley, Western Australia, Australia

**Keywords:** medicine-related burden, medication burden, health-related quality of life, quality of life

## Abstract

Managing multiple medications can be challenging for many patients and negatively affect their health-related quality of life (HRQoL). This study examined the key factors that predict medication burden and HRQoL separately. The secondary aim was to investigate whether overall experience of medication burden mediates the relationship between the number of medications taken and HRQoL. A cross-sectional study was conducted with 348 participants. The average age of these participants was 31 years (SD ± 11.3 years). The average number of medications used was 3.2 (SD ± 1.6). The majority of the study participants experienced moderate (39.0%) to high (45.0%) levels of medication burden. Limited autonomy in adjusting their regimen and concerns about their medications were identified as major contributors to this burden. Hierarchical linear regression analysis indicated that overall experience of medication burden, self-rated health, and a diagnosis of diabetes mellitus significantly predicted medication burden. Similarly, self-rated health, dosing frequency, being female, and assistance with medicines were significant predictors of poor HRQoL. Mediation analysis revealed that overall experience of medication burden partially mediated the association between number of medicines used and HRQoL. These findings highlight the importance of addressing medication burden when developing interventions to avoid jeopardizing patients’ HRQoL. Healthcare professionals should prioritize understanding the experiences of nonelderly patients with their medications and personalize treatment plans accordingly.

## Introduction

Chronic conditions or long-term health conditions are the biggest contributors to global disease burden.^1^ The 2022 National Health Survey revealed an emerging problem with about 81.4% of Australians of all ages living with at least one chronic condition.^2^

Prescription medications are the primary healthcare intervention for chronic conditions aimed at treatment and mitigation of symptoms exacerbation over time.^3^ Moreover, the prescription of multiple medications is a norm in healthcare. The 2022 data from the Pharmaceutical Benefits Scheme reported that Australians over the age of 18 accounted for more than 90% of medicine use in Australia, with an average of 4.4 medications per person.^4,5^ Medication use increases with age, with an average of 8 medications reported for those aged 75 and above.^5^

A growing body of literature indicates that many patients taking multiple medications do not manage their medicines effectively.^6–9^ Patients can experience the burden of medicines at any point in time of their medication journey but limited insights into this burden are available at this stage.^8,10^ Medication burden is multifaceted^6^; it encompasses a wide range of contributing factors such as the complexity of the medicine regimen, financial concerns, and interference in their daily lives.^6–8^

A meta-synthesis by Mohammed et al^8^ found that the burden of medicines experienced by patients influences their future medicine-related beliefs and practices, which directly impacts their psychosocial, physical, and financial well-being. A positive experience leads to better disease control and a positive relationship, whereas adverse events are associated with poor disease control. Therefore, an important consideration in any treatment is that medications should not interfere with patients’ health-related quality of life (HRQoL). This is the principle of “minimally disruptive medicine.” However, the existing literature has reported that patients taking multiple medications tended to experience deterioration in their HRQoL.^11–15^

Health-related quality of life is an important patient-reported outcome that reflects the effectiveness of treatment from the patients’ perspective. Several studies have examined factors associated with HRQoL in individuals taking multiple medicines.^8,11,13–16^ Notably, the Australian study conducted by Chen et al^11^ showed that patients who experienced a higher medication burden tended to have lower HRQoL.

The existing literature has primarily focused on understanding the burden of medicine in older adults with polypharmacy.^7,11,17^ However, a significant body of evidence consistently demonstrates that nonelderly individuals who use relatively fewer medications experience greater medication burden in comparison to elderly individuals who show greater acceptance of their medications.^7,18,19^ Therefore, further investigation into nonelderly adults’ medication burden experience and its impact on their HRQoL is warranted. This information is critical in the development of customized interventions aimed at not only minimizing patients’ medication burden but also optimizing their HRQoL.

Hence, the primary aim of the current study was to identify factors that predict medication burden and HRQoL separately. The secondary objective was to determine whether the experience of overall medication burden mediates the relationship between increase in number of medicines used and HRQoL.

## Materials and Methods

### Study Design and Participants

A cross-sectional study was conducted at Swinburne University of Technology with approval from the institutional research ethics committee (Ref: 20211119-6786). Participants who were aged 18 years or older and took two or more prescription medications concurrently were recruited through convenience sampling via advertisements at this university.

### Research Questionnaire

After appropriate written informed consent, participants were directed to spend about 60 min completing a survey using the online Qualtrics platform. The research instrument for this survey consisted of three sections: (1) Living with Medicine Questionnaire version 3 (LMQ-3), (2) EQ-5D-5L questionnaire, and (3) Demographic background. Permission to use the LMQ-3 (UK English version) was obtained from the University of Greenwich and Kent at Medway, and the EuroQol Research Foundation at Rotterdam granted permission to use the EQ-5D-5L (UK English version).

#### Living with Medicines Questionnaire Version 3

Medication burden was measured using the third version of the Living with Medicines questionnaire (LMQ-3). The LMQ-3 has been shown to be a reliable and valid measure of medication burden in individuals who use multiple medications for their chronic conditions.^6^

The LMQ-3 consists of 8 domains and 41 items scored on a 5-point Likert scale (1 = strongly agree, 2 = agree, 3 = neutral, 4 = disagree, 5 = strongly disagree). The eight domains comprise practical difficulties, interference with daily life, patient–clinician communication, cost-related burden, autonomy, lack of effectiveness, concerns about medicines, and side effects.^6^ The items from each domain are summed to calculate a composite LMQ-3 score, which ranges from 41 to 205, with a higher score indicating a greater medication burden. Participants who scored 41 to 87, 99 to 110, and 111 to 205 were considered to have low, moderate, and high levels of medication burden, respectively.^6^

The LMQ-3 also consists of a single item visual analog scale (VAS), which measures participants’ overall experience of all their prescribed medicines on a scale of 0 to 10. The LMQ VAS categorizes overall medication burden as no/minimal burden = 0 to 4, some degree of burden = 4.1 to 5.9, and a high degree of burden = 6 to 10.^6^

#### EQ-5D-5L Questionnaire

Health-related quality of life was measured using the EQ-5D-5L questionnaire. The EQ-5D-5L is extensively used and deemed to be a reliable and valid measure of HRQoL. Five dimensions, mobility, self-care, usual activities, pain/discomfort, and anxiety/depression, were measured across five levels (1 = no problems, 2 = slight problems, 3 = moderate problems, 4 = severe problems, 5 = extreme problems).^20^

The EQ-5D-5L identifies 3215 distinct health states that can be converted into a single summary utility score based on the general population to generate a health profile for each participant. The utility scores generated by the EQ-5D-5L range from 0 (death) to 1 (perfect health).^20^ Negative scores indicate the perceived health states are worse than death.^20^ Although the Australian population value set has been developed, the scoring algorithm is not yet available at this stage.^21^ Hence, this study obtained utility scores by using the scoring algorithm from the United Kingdom, with scores ranging from −0.285 to 1.^22^

The second component of this questionnaire is a single-item VAS (EQ-5D-5L VAS) to measure the respondent's current health status with endpoints of 0 (worst health) and 100 (best health).^23^

#### Demographic characteristics

The last part of the research instrument captured the respondent's demographic background (such as age, gender, employment status, education, nationality, self-rated health condition, and history of selected chronic conditions including hypertension, diabetes mellitus, heart problems, stroke, cataract/glaucoma, hearing problem, arthritis, and dementia), as well as medication characteristics (such as the number of medicines, medicine formulation type, dosing frequency, paying for prescription, and medicine assistance).

### Statistical Analysis

Descriptive statistics (such as mean, standard deviation median, and range) were presented for continuous variables, while frequencies and percentages were reported for categorical variables. Given the substantial deviation from normality, Spearman rank correlation coefficient was used to quantify the strength and direction of the linear relationship between study variables.

Two hierarchical multiple linear regressions using stepwise elimination procedure were performed separately to systematically evaluate the impact of different sets of predictors on medication burden and HRQoL while controlling for covariates such as the number of medicines, age, and the number of chronic conditions. Hierarchical multiple linear regression analyses were conducted in two steps. In the first step, covariates were entered into the model followed by the inclusion of significant predictors into the second model. All relevant diagnostics for multiple linear regression analysis (including normality, multicollinearity, outliers, influential points, and linearity) were evaluated to ensure the validity of the analysis.

Next, a simple mediation model was conducted by employing Hayes PROCESS macro^24^ (version 4.2) model 4 to evaluate the mediating effect of the overall experience of medication burden in the relationship between the number of medications and HRQoL. Age and the number of chronic conditions were entered into the mediation model as covariates.

A *P* value of <.05 was considered statistically significant for all 2-sided tests. All missing data were excluded from the analysis, performed using IBM SPSS Statistics version 29 (IBM Corp.).

## Results

### Descriptive Statistics

A total of 348 participants completed this study. Participants were aged from 18 to 78 years old, with an average age of 31.0 years (SD ± 11.3 years). Participants were predominantly female (79%), Australian (74%), have completed tertiary education (61%), and were employed either full time or part time (53%). Most of the participants rated their health as moderate (27%) to good (58%)(Table 1).

**Table 1.** Demographics and Medicine-Related Characteristics.

Demographic characteristics	All participants (n* *= 348)
Age (years)	
Median (range)	38 (18-78)
Mean ± SD	31.0 ± 11.3
Gender, n (%)	
Female	275 (79%)
Male	73 (21%)
Nationality, n (%)	
Australian	259 (74%)
Non-Australian	89 (26%)
Highest educational attainment, n (%)	
Primary/Secondary	135 (39%)
Tertiary	213 (61%)
Employment status, n (%)	
Employed (part time/full time)	183 (53%)
Unemployed	13 (4%)
Student (part time/full time)	119 (34%)
Retired	1 (0.3%)
Other	32 (9%)
Self-rated health condition, n (%)	
Excellent	29 (8%)
Good	203 (58%)
Moderate	93 (27%)
Poor	23 (7%)
Number of chronic conditions, n (%)	
0	204 (59%)
1	94 (27%)
≥2	50 (14%)
Diagnosis of chronic condition(s),^a^ n (%)	
Hypertension	57 (16%)
Diabetes mellitus	14 (4%)
Heart problems	31 (9%)
Stroke	4 (1%)
Cataract / glaucoma	8 (2%)
Hearing problem	20 (6%)
Arthritis	77 (22%)
Dementia	1 (0.3%)
Assistance with medicines, n (%)	
Yes	55 (16%)
Paying for prescription, n (%)	
Yes	326 (94%)
Number of medicines	
Median (range)	3 (2-13)
Mean ± SD	3.2 ± 1.6
Formulation of medicines,^a^ n (%)	
Tablet/capsule	238 (68%)
Others	9 (3%)
Both (tablets/capsules with other formulation)	101 (29%)
Dosing frequency,^a^ n (%)	
Sometimes/when needed	6 (2%)
Once a day	206 (58%)
Twice a day	102 (29%)
Thrice a day	30 (9%)
More than 3 times a day	8 (2%)

^a^ Participants were allowed to select multiple responses.

The number of concurrent medications prescribed to participants ranged from 2 to 13, with an average of 3.2 (SD ± 1.6). Almost all participants (94%) paid for their prescriptions and independently managed their medications (84%). About 59% of the participants had no chronic conditions, 68% of the participants used oral solid formulations, and 58% used their medicines once daily.

### Correlation Between LMQ-3 Scores and EQ-5D-5L Scores

The composite LMQ-3 scores were moderately negatively associated with EQ-5D-5L utility scores (*r *= −0.432, *P *< .001), as well as weakly negatively associated with the EQ-5D-5L VAS scores (*r *= −0.302, *P *< .001). A similar weak negative relationship was observed between EQ-5D-5L VAS scores and LMQ VAS scores (*r *= −0.277, *P *< .001), as well as between EQ-5D-5L utility scores and LMQ VAS scores (*r *= −0.350, *P *< .001) (Supplemental Table 1).

### Living with Medicine Questionnaire-3 Domain Scores (Key Mechanisms of Medication Burden)

According to the LMQ-3 domain score analysis results summarized in Supplemental Table 2, the top two key mechanisms of medication burden were autonomy to vary regimens (66%) and concern about medicines (62%).

### Regression Analysis

Table 2 shows that Model 1 explained about 2.8% of the variance in medication burden measured by composite LMQ-3 (*F*_(3, 344)_ = 4.32, *P *= .005). After adjusting for confounding variables, the regression analysis results summarized in Model 2 shows that overall experience of medication burden measured by LMQ VAS, self-rated health, and living with diabetes mellitus accounted for 47.6% of the variance in composite LMQ-3 (*F*_(6, 341)_ = 54.82, *P *< .001) (Table 2). Overall, LMQ VAS (β = 0.62, *P *< .001), self-rated health (β = 0.16, *P *< .003), and having a diagnosis of diabetes mellitus (β = −0.15, *P *= .001) were significant in predicting medication burden.

**Table 2.** Hierarchical Regression Model Identifying Significant Factors That Predict Medicine-Related Burden (Composite LMQ-3 Score).^a^

Model	Variable	B	SE	β	t	Sig.	Model summary
1	Constant	114.03	3.50		32.55	<.001	*R*^2 ^= 0.036, Adj. *R*^2 ^= 0.028, *F* = 4.32, *P *= .005
	Number of medicines	0.94	0.68	0.08	1.38	.168	
	Age (years)	−0.33	0.10	−0.18	−3.31	.001	
	Number of chronic conditions	2.07	1.28	0.09	1.63	.105	
2	Constant	84.34	6.15		13.72	<.001	*R*^2 ^= 0.506, Adj. *R*^2 ^= 0.476, *F* = 16.75, *P* < .001
	Number of medicines	−0.68	0.58	−0.06	−1.16	.246	
	Age (years)	−0.24	0.09	−0.14	−2.73	.007	
	Number of chronic conditions	4.85	2.13	0.21	2.28	.023	
	LMQ VAS (overall medication burden)	5.28	0.35	0.62	14.89	<.001	
	Self-rated health	4.58	1.19	0.16	3.84	<.001	
	Diabetes mellitus	−15.61	4.79	−0.15	−3.26	.001	

Abbreviations: B, unstandardized regression coefficient; β, standardized coefficient beta; LMQ-3, Living with Medicine Questionnaire version 3; SE, coefficient standard error; VAS, visual analog scale.

^a^ Categorical variables were binary coded. Diabetes mellitus (No = 0, Yes = 1).

In Table 3, the first model explained about 5.7% of the variance in HRQoL, measured by EQ-5D-5L VAS (*F*_(3, 344)_ = 8.01, *P *< .001). After adjusting for confounding variables, the overall regression model (Model 2) explained about 45.2% of the variance in HRQoL. The same model subsequently revealed that factors such as self-rated health (β = −0.59, *P *< .001), dosing frequency (β = −0.15, *P *< .001), of female gender (β = −0.11, *P *= .008), and requiring assistance with medicine (β = −0.11, *P *= .012) were significant in predicting HRQoL.

**Table 3.** Hierarchical Regression Model Identifying Significant Factors That Predict Health-Related Quality of Life (EQ-5D-5L VAS).^a^

Model	Variable	B	SE	β	t	Sig.	Model summary
1	Constant	68.18	3.25		20.98	<.001	*R*^2 ^= 0.065, Adj. *R*^2 ^= 0.057, *F* = 8.01, *P* < .001
	Number of medicines	−2.55	0.63	−0.22	−4.06	<.001	
	Age (years)	0.24	0.09	0.14	2.57	.011	
	Number of chronic conditions	−1.91	1.12	−0.09	−1.61	.108	
2	Constant	124.87	5.92		21.09	<.001	*R*^2 ^= 0.484, Adj. *R*^2 ^= 0.452, *F* = 15.33, *P* < .001
	Number of medicines	0.13	0.56	0.01	0.23	.821	
	Age (years)	0.23	0.09	0.13	2.64	.009	
	Number of chronic conditions	−2.39	2.05	−0.11	−1.17	.244	
	Self-rated health	−15.90	1.15	−0.59	−13.84	<.001	
	Dosing frequency	−4.02	1.20	−0.15	−3.34	<.001	
	Gender (female)	−5.21	1.95	−0.11	−2.67	.008	
	Assistance with medicines	−5.82	2.32	−0.11	−2.51	.012	

Abbreviations: B, unstandardized regression coefficient; β, standardized coefficient; SE, coefficient standard error; VAS, visual analog scale.

^a^ Categorical variables were binary coded. Gender (Male = 0, Female = 1), assistance with medicines (No = 0, Yes = 1).

### Mediation Model

**Figure 1.**
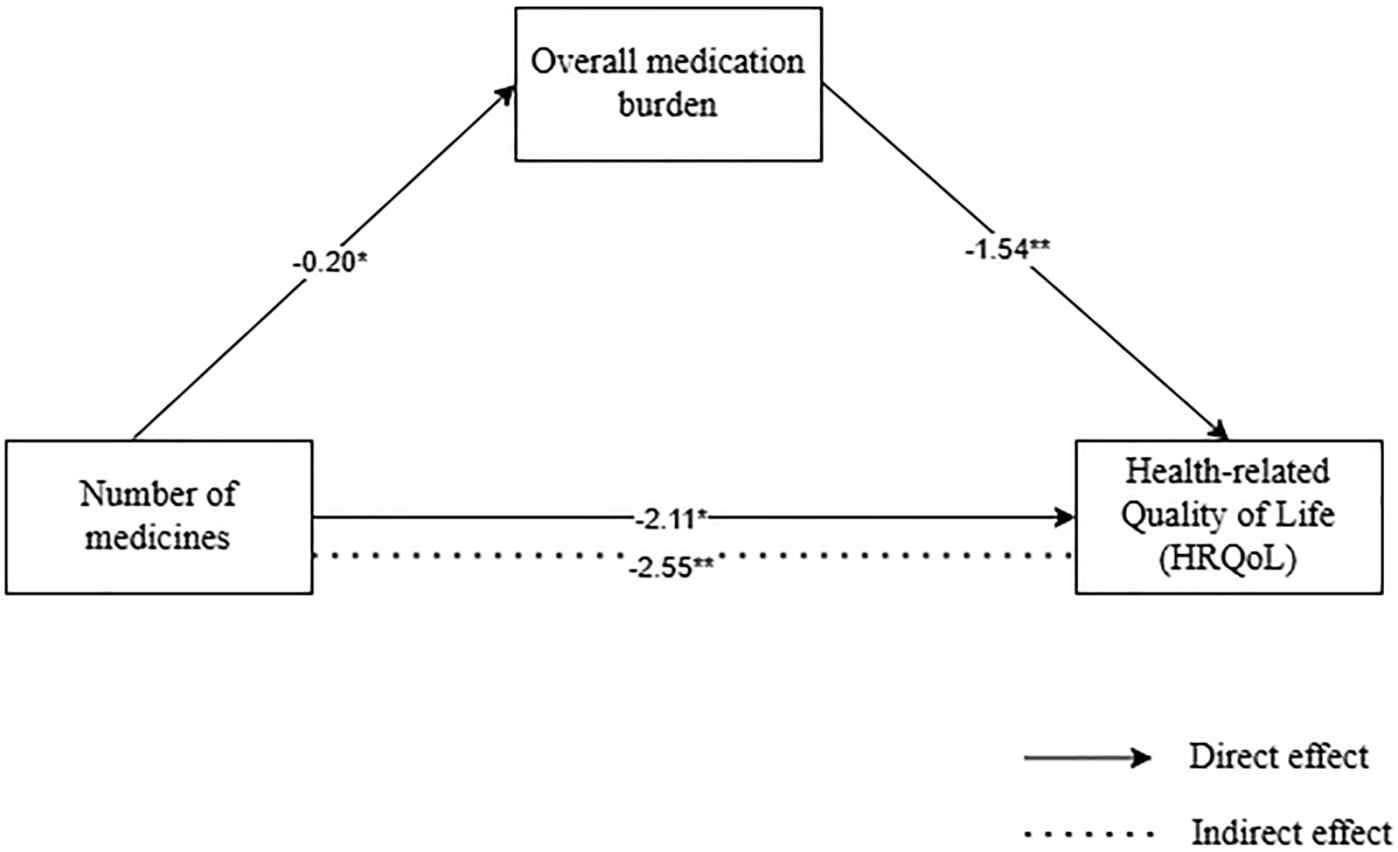
Simple mediation analysis. Unstandardized regression coefficients are reported in the figure. Direct and indirect effects of number of medicines on health-related quality of life (HRQoL). *P < .01, **P < .001.

After controlling for covariates, the mediation model was found to be significant in explaining approximately 10.1% of the variance in HRQoL (*F*_(4, 343)_ = 9.64, *P *< .001). The number of medicines used directly predicted HRQoL when excluding the mediator (β = −2.11, *P *< .01). The number of medicines used was a significant predictor of the overall experience of medication burden (β = 0.29, *P* < .001). The direct impact of the number of medicines used on HRQoL remained significant even after considering the mediating factor of the overall experience of medication burden (β = −2.55, *P* < .001). These findings suggest that high medication use indirectly impacted HRQoL, and this relationship was partially mediated by the overall experience of medication burden. Additionally, the bootstrap confidence interval (based on 5000 samples) indicated the significant indirect effect of the mediation model (95% CI = [−0.94, −0.11]) (Figure 1).

## Discussion

The present study revealed that most participants experienced moderate (39.0%) to high (45.0%) levels of medication burden. Medication use is complex and shaped by the interplay of medication characteristics and psychosocial factors.^6–8^ These elements influence how patients perceive their medications and the associated burden. Previous studies have demonstrated that as the number of medications increases, the burden on patients is likely to intensify due to the cognitive and physical effort needed to manage them.^6–8^

The results in the current study were higher than those reported in a study by Chen et al.^11^ which showed that older Australian adults with polypharmacy who experienced minimal (26.9%) to moderate (44.5%) levels of medication burden.^11^ This study showed nonelderly adults experience higher medication burden despite using relatively fewer medicines compared to their elderly counterparts.^7,18^ Previous studies have revealed that adults are likely to struggle with incorporating medications into their daily lives as they juggle their social responsibilities, commitments, and relationships.^8,18^ Furthermore, the stigma associated with the use of medications at a young age is associated with negative emotions regarding medication use.^25^ In contrast, studies have shown that elderly patients display greater psychological resilience and are more accustomed to using medicines.^7,18,19^

The levels of medication burden observed in our study also varies from the levels of burden reported in previous studies including minimal (35.4%) to moderate (62.0%) levels in Kuwait^17^; minimal (24.4%) to moderate (45.1%) in New Zealand^18^; minimal (66.8%) to moderate (24.1%) in Qatar^26^; and minimal (33.1%) to moderate (54.6%) in the United Kingdom.^7^ The observed differences across countries highlight the impact of cultural differences on medication burden. Cultural health beliefs and practices may impact how individuals perceive their medications.^27^ A study performed by Horne et al^27^ found that people of Asian origin had a more negative attitude toward medicines than those of European descent. Further exploration surrounding the influence of cultural background on the perception and experience of medication may be an essential point of enquiry.^28^

Consistent with a previous study, medication burden and overall experience of medication burden were found to be negatively associated with HRQoL.^11^ This study highlights the importance of medication management, which includes consultation with health practitioners, medication purchasing, and dealing with the stigma associated with medicine use.^6–9^

The majority of participants (84%) reported problems with the anxiety/depression aspects of the HRQoL. A previous study conducted based on the Australian population revealed that the prevalence of anxiety and depression issues was higher among nonelderly individuals, and this prevalence was negatively associated with age.^29,30^

The psychosocial impact of the coronavirus disease 2019 (COVID-19) pandemic may have been a contributing factor. In the global increase in psychological distress and a worsening of preexisting mental health issues, Australia experienced the longest and strictest lockdown during the COVID-19 pandemic.^31^ The COVID-19 restrictions on access to nonemergency healthcare and personal consultations may have had an impact on HRQoL.^32^ Difficulty in obtaining personal medical advice may have increased anxiety among individuals struggling to manage their medications.^33^ Social isolation and disruption of daily life were the likely contributors to mental stress and could explain the higher levels of anxiety/depression observed among the participants.^34^

Given the significant association of increasing age with the number of medicines and chronic conditions identified in a previous study^11^, we found that the significant factors that predicted medication burden were overall experience of medication burden, self-rated health, and a diagnosis of diabetes mellitus. Likewise, self-rated health, dosing frequency, being female, and requiring medication assistance were significant predictors of HRQoL.

A review by Sav et al^35^ highlighted that patients with diabetes mellitus experience three times the burden of treatment. It is plausible that the progressive nature of diabetes involves complicated long-term management plans that require patients to constantly monitor their blood sugar, take medications, and manage side effects, which leads them to experience a higher perceived medication burden.

In this conceptual framework, it is logical to conclude that a patient's overall experience is significantly associated with the construct of medication burden.^6^ This relationship suggests that the LMQ VAS, a straightforward and efficient single-item measure, can be effectively employed as a screening tool in clinical consultations to rapidly evaluate a patient's experience. A high LMQ VAS score may indicate that a patient is experiencing burden across multiple domains of medication use, warranting a comprehensive assessment using the full LMQ-3 to identify specific areas of concern for tailored care.

Our results confirm those seen in the general population that women tended to experience lower HRQoL and tended to experience a greater burden of chronic conditions such as chronic pain, autoimmune diseases, and other disabilities, which contribute to the deterioration of their HRQoL.^29,30,36,37^ Cultural health norms shape their experiences as they grow older.^38^ Women often experience greater societal disadvantages, lower socioeconomic status, and are subjected to negative stereotypes, all of which can have a significant impact on their perceived HRQoL.^38^

Finally, higher dosing frequency and requiring assistance with medicines were found to be significant predictors of HRQoL. Individuals with complex medication regimes impact daily functioning and subsequently lower HRQoL.^7,9,35^ Patients and physicians may hold different perspectives about medications.^39^ Ensuring a shared decision-making mechanism throughout consultations can not only help patients reduce imbalances in perspectives but also empower them to potentially improve their HRQoL.

Our findings revealed that an increase in medication use is likely to result in more frequent side effects and adverse events potentially making it hard for patients to adhere to their treatment regimens.^3,40^ These factors appear to negatively impact patients’ emotional well-being and overall life satisfaction leading to deterioration in their HRQoL.^3,40^

Our mediation analysis showed that an increase in the number of medications can indirectly affect HRQoL through increasing medication burden. Taking more medications results in more complex medication regimens, which adds demands to patients’ daily lives.^9^ Managing dosages, timings, and instructions can create cognitive strain, stress, and confusion.^9^ Additionally, patients may need to adjust their daily routines to fit around medication times, which can be particularly challenging for nonelderly individuals with busy schedules.^7,8,18^ This ultimately contributes to greater medication burden and reduces HRQoL. Identifying and addressing medication burden for individuals taking multiple medicines could serve as a protective element for their HRQoL.^41^

The overall experience of medication burden alone cannot explain the intricate relationship between increased medication use and HRQoL. Living with multiple medications is a multifaceted issue. It is plausible that other dimensions of medication burden may contribute to the intricate dynamics of this association, warranting further exploration.^6^

The findings of the study have important clinical implications for healthcare professionals. Clinicians should consider a combination of behavioral and educational approaches that reduce the medication burden for nonelderly patients.^42^ For instance, using telehealth, offers nonelderly patients who are technologically proficient a potentially powerful and cost-effective approach to providing personalized patient education and managing medication plans to reduce the medication burden.^43^ Clinicians should aim to simplify medication regimens for patients who may be experiencing medication burden.^44^ Regularly reviewing patients’ medications and deprescribing unnecessary medications can improve patient satisfaction.^44^ Clinicians can improve adherence to treatment by involving pharmacists in pharmacist-led, multicomponent interventions.^45^

A key strength of the current study is the use of reliable, valid, and multidimensional instruments to measure medication burden and HRQoL. This approach allows for the identification of a broad range of issues that contribute to the experience of medication burden and overall health state. Additionally, by focusing on a younger cohort, the study addresses a less commonly explored demographic, highlighting the unique challenges faced by nonelderly individuals in managing multiple medications.

This study had several limitations. Since this was a cross-sectional study, it was impossible to derive a causal relationship. Secondly, no multicultural information was collected in the data, which may limit the generalizability of our findings to Australia's multicultural population. Moreover, given that the number of medicines used was self-reported, there may have been some underreporting of medicine counts. Furthermore, we did not inquire about the specific reasons for participants taking multiple medications. Consequently, it is unclear why participants required multiple medications, particularly given that most denied having any chronic conditions. Finally, the present study was conducted during the COVID-19 pandemic and the impact of the pandemic on study findings remains unknown. Future research should focus on utilizing a longitudinal study design to better understand the profiles of patients. Additionally, it would be beneficial to gain insights into why participants are using their medications. Furthermore, additional information on Australia's multicultural context would be helpful to better inform future policies.

## Conclusion

Our results suggest that nonelderly adults with relatively fewer medications tended to experience a greater medication burden and a deterioration in their perceived HRQoL compared with older patients. Patients who perceive a greater overall medication burden experience poor health. Patients living with diabetes mellitus, poor health, taking medication frequently, female gender, and requiring assistance with medications are at risk for poor HRQoL. The findings of this study highlight the need for clinicians to consider the medication burden in nonelderly patients in order to improve their HRQoL.

## Supplemental Material

sj-docx-1-jpx-10.1177_23743735251330353 - Supplemental material for Experience of the Burden of Using Multiple Medicines and the Associated Impact on Health-Related Quality of Life Supplemental material, sj-docx-1-jpx-10.1177_23743735251330353 for Experience of the Burden of Using Multiple Medicines and the Associated Impact on Health-Related Quality of Life by Aastha Gurung, Edward Ogden and Won Sun Chen in Journal of Patient Experience
