# Supplementary material for: Experience of the Burden of Using Multiple Medicines and the Associated Impact on Health-Related Quality of Life
Source: J Patient Exp. 2025 Mar 21;12:23743735251330353. doi: 10.1177/23743735251330353 (PMC11930472; doi:10.1177/23743735251330353)
Supplement: sj-docx-1-jpx-10.1177_23743735251330353 - Supplemental material for Experience of the Burden of Using Multiple Medicines and the Associated Impact on Health-Related Quality of Life [file sj-docx-1-jpx-10.1177_23743735251330353.docx]

**Supplementary Table 1**

*Spearman’s Correlation Coefficient Among Study Variables.*

|  | **Variable** | **1** | **2** | **3** | **4** | **5** | **6** | **7** | **8** | **9** | **10** |
| --- | --- | --- | --- | --- | --- | --- | --- | --- | --- | --- | --- |
| **1** | Age | - |  |  |  |  |  |  |  |  |  |
| **2** | Education levels | 0.523^***^ | - |  |  |  |  |  |  |  |  |
| **3** | No. of Medicines | 0.124^*^ | -0.023 | - |  |  |  |  |  |  |  |
| **4** | Dosing frequency | 0.089 | 0.150^**^ | 0.314^***^ | - |  |  |  |  |  |  |
| **5** | No. of chronic conditions | 0.254^***^ | 0.070 | 0.168^**^ | 0.135^*^ | - |  |  |  |  |  |
| **6** | Self-rated health | 0.020 | -0.014 | 0.228^***^ | 0.083 | 0.147^**^ | - |  |  |  |  |
| **7** | Composite LMQ-3 scores | -0.110^*^ | -0.030 | 0.104 | 0.055 | 0.056 | 0.306^***^ | - |  |  |  |
| **8** | LMQ VAS scores | -0.010 | 0.007 | 0.229^***^ | 0.184^***^ | 0.031 | 0.255^***^ | 0.644^***^ | - |  |  |
| **9** | EQ-5D-5L utility scores | 0.178^**^ | 0.087 | -0.228^***^ | -0.167^**^ | -0.167^**^ | -0.471^***^ | -0.432^***^ | -0.350^***^ | - |  |
| **10** | EQ-5D-5L VAS scores | 0.081 | -0.018 | -0.162^**^ | -0.210^***^ | -0.115^*^ | -0.648^***^ | -0.302^***^ | -0.277^***^ | 0.449^***^ | - |

*Correlation is significant at * p<0.05, **p<0.01, ***p<0.001 (2-tailed).*

**Supplementary Table 2**

*LMQ-3 Domain Score Analysis*

|  | Domain | Total item = 41 | Score range | Mean (SD) | Maximum score (%)* |
| --- | --- | --- | --- | --- | --- |
| 1 | Communication with healthcare professionals about medicines | Items = 5 | 5 – 25 | 11.6 (3.6) | 46% |
| 2 | Practical difficulties | Items = 7 | 7 – 35 | 17.8 (4.7) | 51% |
| 3 | Cost-related burden | Items = 3 | 3 – 15 | 7.5 (2.9) | 50% |
| 4 | Side-effect burden | Items = 4 | 4 – 20 | 10.6 (4.1) | 53% |
| 5 | Lack of effectiveness | Items = 6 | 6 – 30 | 14.0 (3.8) | 47% |
| 6 | Concern about medicine use | Items = 7 | 7 – 35 | 21.7 (5.0) | 62% |
| 7 | Interference to day-to-day life | Items = 6 | 6 – 30 | 14.8 (4.8) | 49% |
| 8 | Anatomy to vary regimens | Items = 3 | 3 – 15 | 9.9 (2.7) | 66% |

** Maximum score (%) is estimated according to Tordoff et al.^1^*

1. Tordoff JM, Brenkley C, Krska J, Smith A. Exploring medicines burden among adults in New Zealand: a cross-sectional survey. *Patient preference and adherence*. 2019:2171-2184.
